# Supplementary material for: COZOID: contact zone identifier for visual analysis of protein-protein interactions
Source: BMC Bioinformatics. 2018 Apr 6;19:125. doi: 10.1186/s12859-018-2113-6 (PMC5889581; doi:10.1186/s12859-018-2113-6)
Supplement: Supplementary file 2 — Software build. Executable binary file of the software tool. (ZIP 118,784 kb) [file 12859_2018_2113_MOESM2_ESM.zip › cozoid/java/docs/beansbinding-1.2.1-doc.zip!/org/jdesktop/beansbinding/class-use/AutoBinding.html]

Uses of Class org.jdesktop.beansbinding.AutoBinding (Beans Binding)


---


|  |  |  |
| --- | --- | --- |
| **Overview** \| **Package** \| **Class** \| **Use** \| **Tree** \| **Deprecated** \| **Index** \| **Help** | |  |
| PREV   NEXT | **FRAMES**    **NO FRAMES**     **All Classes** |  |


---


## **Uses of Class org.jdesktop.beansbinding.AutoBinding**

| Packages that use AutoBinding | |
| --- | --- |
| **org.jdesktop.beansbinding** | Provides support for defining properties and creating bindings between sets of two properties. |
| **org.jdesktop.swingbinding** | Provides support for binding to complex Swing components, and documentation on the interesting Swing properties to bind to. |

Uses of AutoBinding in org.jdesktop.beansbinding

| Methods in org.jdesktop.beansbinding that return AutoBinding | |
| --- | --- |
| `static <SS,SV,TS,TV> AutoBinding<SS,SV,TS,TV>` | `Bindings.createAutoBinding(AutoBinding.UpdateStrategy strategy, SS sourceObject, Property<SS,SV> sourceProperty, TS targetObject, Property<TS,TV> targetProperty)`             Creates an instance of `AutoBinding` that binds a property of a source object to a property of a target object. |
| `static <SS,SV,TS,TV> AutoBinding<SS,SV,TS,TV>` | `Bindings.createAutoBinding(AutoBinding.UpdateStrategy strategy, SS sourceObject, Property<SS,SV> sourceProperty, TS targetObject, Property<TS,TV> targetProperty, java.lang.String name)`             Creates a named instance of `AutoBinding` that binds a property of a source object to a property of a target object. |
| `static <SS,TS,TV> AutoBinding<SS,SS,TS,TV>` | `Bindings.createAutoBinding(AutoBinding.UpdateStrategy strategy, SS sourceObject, TS targetObject, Property<TS,TV> targetProperty)`             Creates an instance of `AutoBinding` that binds a source object to a property of a target object. |
| `static <SS,TS,TV> AutoBinding<SS,SS,TS,TV>` | `Bindings.createAutoBinding(AutoBinding.UpdateStrategy strategy, SS sourceObject, TS targetObject, Property<TS,TV> targetProperty, java.lang.String name)`             Creates a named instance of `AutoBinding` that binds a source object to a property of a target object. |

Uses of AutoBinding in org.jdesktop.swingbinding

| Subclasses of AutoBinding in org.jdesktop.swingbinding | |
| --- | --- |
| `class` | `JComboBoxBinding<E,SS,TS>`             Binds a `List` of objects to act as the items of a `JComboBox`. |
| `class` | `JListBinding<E,SS,TS>`             Binds a `List` of objects to act as the elements of a `JList`. |
| `class` | `JTableBinding<E,SS,TS>`             Binds a `List` of objects to act as the rows of a `JTable`. |

---


|  |  |  |
| --- | --- | --- |
| **Overview** \| **Package** \| **Class** \| **Use** \| **Tree** \| **Deprecated** \| **Index** \| **Help** | |  |
| PREV   NEXT | **FRAMES**    **NO FRAMES**     **All Classes** |  |


---
